# Investigating the DTI-ALPS index and its association with cognitive impairment in Alzheimer’s disease: a diffusion tensor imaging study

**DOI:** 10.3389/fnins.2026.1899705

**Published:** 2026-09-08

**Authors:** Zixuan Zhai, Yingte Wang, Hong Li, Yimin Wang, Jing Zhou, Yi Liang, Airong Yang, Zhiming Li

**Affiliations:** 1 Department of Radiology, General Hospital of The Yangtze River Shipping, Wuhan Brain Hospital, Wuhan, Hubei, China; 2 Department of Radiology, The Second Affiliated Hospital, Guangzhou Medical University, Guangzhou, Guangdong, China; 3 Department of Radiology, Guangzhou Eighth People’s Hospital, Guangzhou Medical University, Guangzhou, Guangdong, China; 4 Department of Radiology, The Affiliated Panyu Central Hospital, Guangzhou Medical University, Guangzhou, Guangdong, China; 5 Qinghai Cardio-Cerebrovascular Specialty Hospital, Qinghai High Altitude Medical Research Institute, Xining, Qinghai, China

**Keywords:** Alzheimer’s disease, cognitive level, DTI-ALPS, glymphatic system, TBSS

## Abstract

**Objectives:**

With the global aging population, Alzheimer’s disease (AD) poses a major health challenge. The diffusion tensor image (DTI) analysis along the perivascular space (DTI-ALPS) index has emerged as a noninvasive imaging marker that indirectly reflects glymphatic function. However, its alterations in AD and their associations with cognitive impairment remain incompletely understood. This study primarily aimed to investigate changes in the bilateral ALPS index in patients with AD and to evaluate their associations with cognitive, functional, and emotional measures. Secondary analyses further explored white matter microstructural alterations and their relationships with the ALPS index.

**Materials and methods:**

In total, 35 patients with AD and 21 cognitively normal healthy controls (HC) underwent 3.0T MRI and neuropsychological assessments. Tract-based spatial statistics (TBSS) analysis revealed areas of white matter damage and injured fibers in patients. Bilateral ALPS indices were compared between groups using analysis of covariance (ANCOVA) adjusted for age and gender. TBSS and ROI-based ANCOVA analyses of fractional anisotropy (FA) and mean kurtosis (MK) were performed as secondary analyses. Partial correlation analyses were used to examine associations between ALPS indices, white matter metrics, and neuropsychological scores.

**Results:**

Compared with the HC group, the AD group showed significantly lower bilateral ALPS indices, and these differences remained significant after adjustment for age and gender (left: *F* = 14.404, *P* < 0.001; right: *F* = 28.513, *P* < 0.001). Bilateral ALPS indices were significantly correlated with cognitive and functional measures, including the Mini-Mental State Examination, Montreal Cognitive Assessment, and Activities of Daily Living (ADL), and were also significantly associated with emotional measures, including the Hamilton Anxiety Scale and Hamilton Depression Scale. Among these, the strongest associations were observed with ADL (left: *r* = 0.688; right: *r* = 0.701; both P_FDR < 1 × 10–7). Secondary analyses revealed widespread white matter microstructural alterations in AD. Region-of-interest-based analyses showed that the left hippocampal cingulum exhibited significant differences in both FA and MK, and these metrics were positively associated with the ALPS index as well as with cognitive and functional performance.

**Conclusion:**

The glymphatic system of AD patients has a reduced ability to clear waste. The ALPS index was closely associated with cognitive, functional, and emotional status, while white matter abnormalities, particularly in the left hippocampal cingulum, may represent a structural correlate of glymphatic dysfunction in AD.

## 1 Introduction

**FIGURE 1:**
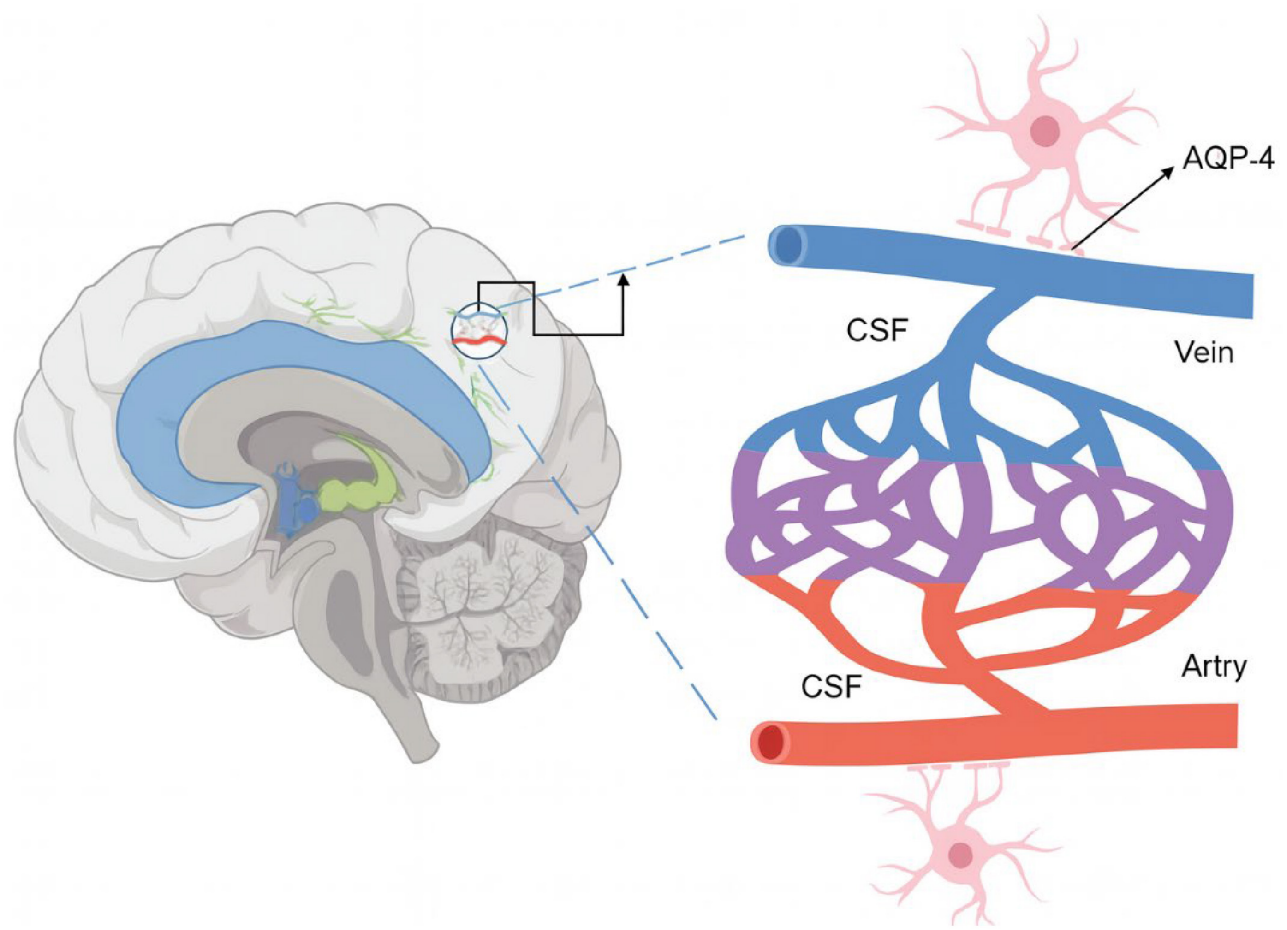
This diagram is an example plot of lymphatic system clearance of metabolites.

Dementia comprises a group of neurological disorders characterized by progressive memory loss and cognitive decline. Alzheimer’s disease (AD) is the most common form of dementia, accounting for approximately 50%–70% of cases. Early manifestations typically include difficulty remembering recent events, while disease progression is often accompanied by disorientation, mood changes, confusion, worsening memory impairment, behavioral abnormalities, and difficulties in speech, swallowing, and walking (Winblad et al., 2016). The glymphatic system is thought to facilitate the clearance of metabolic waste from the brain through the exchange of interstitial fluid and cerebrospinal fluid (CSF), a process promoted by aquaporin-4 expressed on astrocytic end-feet. Together with the blood-brain barrier, the glymphatic system is considered an important pathway for the clearance of amyloid β-protein (Aβ) from the brain (Benveniste et al., 2019). Glymphatic system transportation disorders are believed to be related to chronic brain injury inflammatory mediators and various neurological diseases, including AD, particularly the glymphatic system’s ability to clear brain metabolites (Reeves et al., 2020). Moreover, glymphatic system dysfunction is associated with the accumulation of tau protein and Aβ, which is considered to be the basis of the pathogenesis of AD (Rasmussen et al., 2018; Wu et al., 2021). The glymphatic pathway involved in brain metabolite clearance is schematically illustrated in Figure 1 below.

In the diagnosis of AD, positivity for tau and Aβ is generally regarded as an important pathological criterion (Sullan et al., 2018). Since 2017, diffusion tensor image (DTI) analysis along the perivascular space (DTI-ALPS) has emerged as a noninvasive imaging approach for indirectly evaluating glymphatic function in the brain (Taoka et al., 2017). In recent years, the ALPS method has also been applied to other neurodegenerative and vascular conditions, including Parkinson’s disease, cerebrovascular disease, and type 2 diabetes (McKnight et al., 2021; Toh and Siow, 2021). This technique estimates water diffusivity along the direction of the perivascular space. At the level of the lateral ventricular body, the perivascular space is assumed to run perpendicular to the ventricular wall in the left–right direction, defined as the *x*-axis. In this plane, projection fibers predominantly extend in the head–foot direction, whereas association fibers mainly extend in the anterior–posterior direction, corresponding to the y- and z-axes, respectively. Based on this anatomical arrangement, the ALPS index is defined as the ratio of the mean diffusivity along the *x*-axis in the projection fiber area (Dxxproj) and the diffusivity along the *x*-axis in the association fiber area (Dxxassoc) to the mean diffusivity along the *y*-axis in the projection fiber area (Dyyproj) and the diffusivity along the *z*-axis in the association fiber area (Dzzassoc) (Taoka et al., 2017).

**FIGURE 2:**
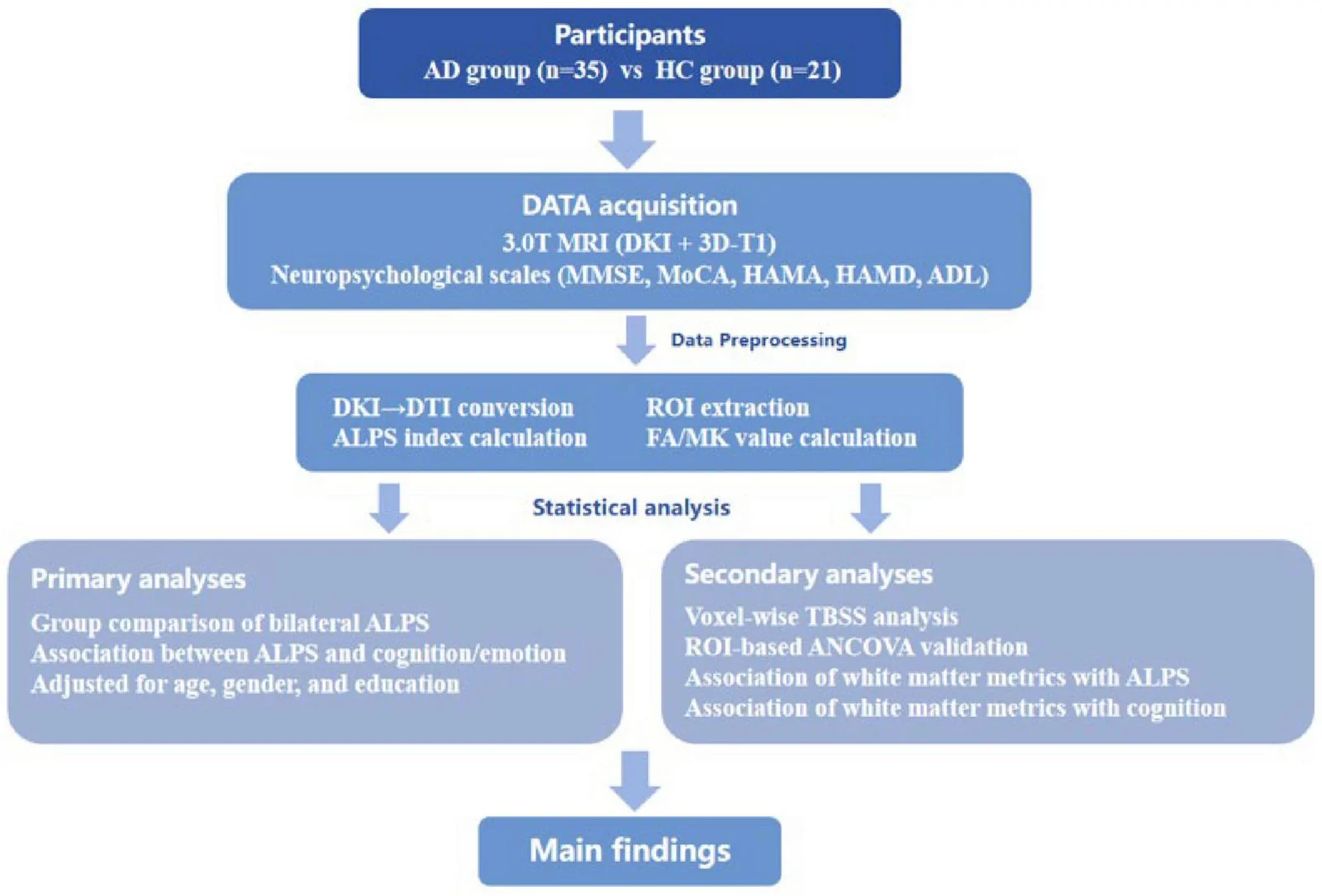
Overall study workflow. Flowchart summarizing participant enrollment, clinical and MRI assessment, image processing, primary analyses of bilateral DTI-ALPS indices, and secondary analyses of white matter microstructure.

Despite growing interest in the ALPS index, research in patients with AD remains limited. In particular, it is still unclear whether glymphatic dysfunction, as reflected by the ALPS index, is associated with white matter microstructural injury and clinical impairment in AD. Therefore, this study aimed to investigate changes in the bilateral DTI-ALPS index in patients with AD and to further explore its relationships with white matter microstructural alterations and cognitive, functional, and emotional measures. The overall study design and analytical framework are summarized in Figure 2.

## 2 Patients and methods

### 2.1 Participants

We included 35 AD patients and 21 cognitively normal healthy controls (HC). This study was approved by the institutional ethics committee, and all participants or their legal guardians provided written informed consent in accordance with the Declaration of Helsinki and its amendments.

All participants underwent neuropsychological assessments, medical history inquiries, brain MRI, and PET-CT or CSF examinations. AD was diagnosed according to the 2024 NIA-AA criteria, requiring positivity for both Aβ and phosphorylated tau. Aβ positivity was defined as CSF Aβ42 < 550 pg/mL or a PET standard uptake value ratio >1.1; tau positivity as CSF p-tau181 > 60 pg/mL. HC participants had negative Aβ and tau biomarkers and an MMSE score ≥27.

Inclusion criteria for the AD group: (1) aged 47–85 years; (2) right-handed, normal auditory and visual functions; (3) pathological diagnosis of AD according to the 2024 NIA-AA guidelines; (4) able to complete cognitive assessment tests independently, with MMSE < 27; (5) completed T1-weighted imaging (T1WI) and diffusion kurtosis imaging (DKI) MR imaging; (6) informed consent signed by the participant or their guardian; (7) no other known brain diseases. Inclusion criteria for the HC group: (1) aged 46–83 years; (2) right-handed, normal auditory and visual functions; (3) negative AD biomarkers (PET-CT or CSF); (4) MMSE ≥ 27; (5) completed T1WI and DKI MR imaging. Exclusion criteria for both groups: (1) primary non-memory cognitive impairment; (2) severe psychiatric or behavioral abnormalities; (3) inability to cooperate with cognitive assessments; (4) history of neurosurgery; (5) prior brain radiotherapy; (6) carbon monoxide poisoning, alcoholism, or metabolic brain injuries.

All participants underwent routine brain MRI; those with any other abnormalities were excluded. Neuropsychological assessments were performed by trained neurologists within 24 h before the MRI scan.

### 2.2 Clinical and laboratory data

Neuropsychological assessments were performed using the following scales. Cognitive function was evaluated using the Mini-Mental State Examination (MMSE; range 0–30, higher scores indicate better cognitive function) and the Montreal Cognitive Assessment (MoCA; range 0–30, higher scores indicate better cognitive function). Anxiety and depressive symptoms were assessed with the Hamilton Anxiety Scale (HAMA; range 0–56, higher scores indicate more severe anxiety) and the Hamilton Depression Scale (HAMD; range 0–52, higher scores indicate more severe depression). Activities of daily living were measured with the Barthel Index (ADL; range 0–100, higher scores indicate greater independence and better functional performance). The researchers evaluating the neuropsychological scales in the study were all trained and consistently assessed neurologists.

### 2.3 MRI data acquisition

MRI data for all subjects were collected using a 3.0 T MR scanner (Philips, Ingenia) with a 32-channel orthogonal head coil, including axial FFE T1 sequences and axial DKI sequences [15 directions, 3 gradients (0, 1000 s/mm 2, 2000 s/mm 2)]. As described in Table 1,DKI images were acquired with the following parameters: echo time (TE) = 0.11 s, repetition time (TR) = 3.33 s, field of view (FOV) = 100, Acquisition Matrix PE = 87, Recon Matrix PE = 224,slice thickness = 3 mm, Spacing Between Slices = 3 mm, Flip Angle = 90°. High-resolution 3D-T1-weighted structural images were acquired using a 3D gradient-echo sequence with TE = 0.02 s, TR = 0.01 s, FOV = 88.28, Acquisition Matrix PE = 230, Recon Matrix PE = 512, slice thickness = 1 mm, Spacing Between Slices = 1 mm, flip angle = 8.

**TABLE 1:** The basic parameters of the main scan sequences.

Parameters	T1WI	DKI
Slice thickness	1 mm	3 mm
Spacing Between Slices	1 mm	3 mm
Echo time	0.02 s	0.11 s
Repetition time	0.01 s	3.33 s
Flip angle	8°	90°
Percent phase FOV	88.28	100
Acquisition Matrix PE	230	87
Recon Matrix PE	512	224

### 2.4 DKI image preprocessing and DTI-ALPS value calculation

Before preprocessing, all DKI images were visually inspected by an experienced neuroradiologist to remove images with significant visual noise and motion artifacts, resulting in the exclusion of one healthy control group patient due to pronounced motion artifacts.

Using the dcm2niix tool to convert 4D DKI DICOM file format to NIFTI file format, we created a bash script to calculate the ALPS value by inputting DKI data through a FOR loop.

The specific steps include splitting the *b*-values in DKI and selecting *b* = 1000 s/mm^2^ to form the DTI image. Using the MRtrix3 command line “dwisenoise” and “mrdegibbs” to remove Gibbs artifacts and eliminate abnormal noise, and performing eddy current correction and motion artifact correction using the FSL command line “topup” in conjunction with “eddy.” Unlike most previous articles, this correction process additionally uses the command line “biascorrect” to correct for bias fields caused by magnetic field inhomogeneity during the MR scanning process to eliminate this influence. Use the FSL command line “dtifit” to generate fractional anisotropy (FA) maps and the *x*-axis, *y*-axis, and *z*-axis diffusion rates. The generated FA maps for each subject are registered and aligned to the standard template JHU-ICBM-FA-1 mm. In FSL, the matrix is applied to the diffusion maps using the command lines “flirt-ref” and “fnirt-ref.” Based on previous studies and methodological consensus, the projection and association fiber regions at the level of the lateral ventricles were identified in the superior corona radiata and superior longitudinal fasciculus, respectively, according to the JHU-ICBM-DTI-81 white matter atlas (Liu et al., 2024). Spherical ROIs with a diameter of 5 mm were automatically placed bilaterally in these regions. Diffusivities along the x-, y-, and z-axes were then measured bilaterally, and the ALPS index was calculated according to the anatomical orientation of the perivascular space, projection fibers, and association fibers, as follows:


$$\mathrm{DTI}-\mathrm{ALPS}=\frac{\mathrm{mean}\,\left(\mathrm{Dxxproj},\mathrm{Dxxassoc}\right)}{\mathrm{mean}\,\left(\mathrm{Dyyproj},\mathrm{Dzzassoc}\right)}$$ (1)

**FIGURE 3:**
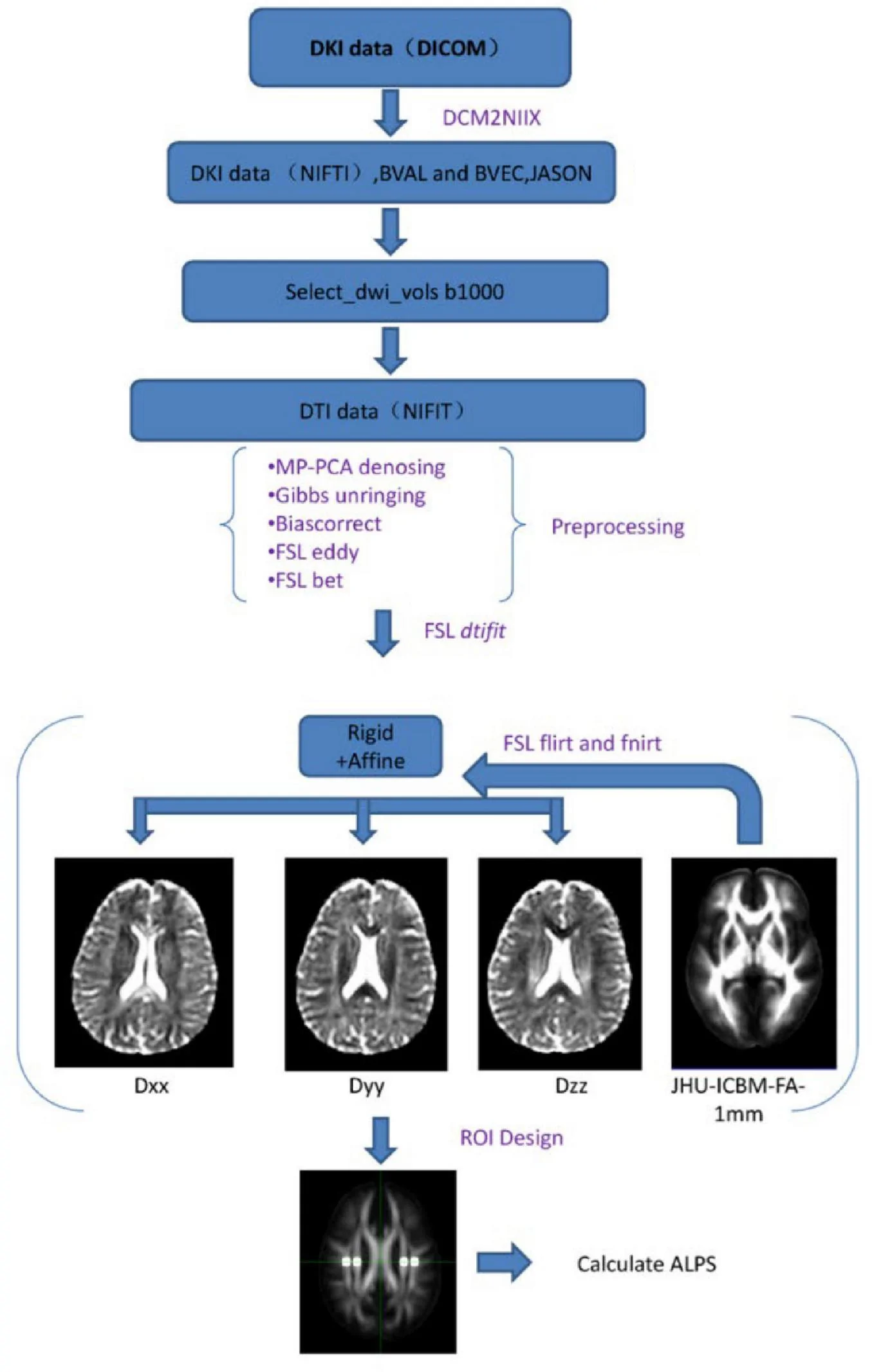
Flowchart of image preprocessing and analysis.

The DKI raw data converted to NIFTI format is subjected to eddy current correction using the “eddy” command. The gradient direction file (BVEC) is regenerated using the “fdt” command. The b0 images of each subject are extracted using the “fslroi” command, and the generated b0 images are skull-stripped and scalp-stripped using the “bet” command. Finally, the processed DKI data is obtained by multiplying the mask of the b0 image with the corrected images. Use the DKE toolkit to fit the preprocessed images to generate FA, mean diffusivity, axial diffusivity, and radial diffusivity values, as well as mean kurtosis, axial kurtosis, radial kurtosis, and kurtosis fractional anisotropy. Mean kurtosis (MK) is the most representative parameter of DKI, defined as the average kurtosis across all directions, and is considered an indicator of the complexity of tissue microstructure. During the TBSS process, the FA and MK images of each participant were registered to the FMRIB58_FA template. By using an FA threshold of 0.2, the average FA image was calculated from all participants’ images, generating an average FA skeleton. Each subject’s FA image was projected onto the skeleton. Finally, the MK skeleton was created using the projection parameters obtained in the previous steps. Due to the complex structure of the human brain, strictly speaking, the diffusion of water molecules is non-Gaussian. Therefore, using DKI instead of DTI can better simulate the true conditions of the human brain. Moreover, DTI only reflects the reversibility; the higher the kurtosis, the more significantly the non-Gaussian diffusion of water molecules is restricted. DKI parameters can also better reflect the complex directional interwoven distribution of neural fiber structures. TBSS analyzes only the voxels closest to the white matter fiber skeleton, which can effectively reduce statistical errors caused by imperfect registration. The overall image preprocessing and analysis pipeline is summarized in Figure 3.

### 2.5 Statistical analysis

The statistical analysis of all subjects was based on SPSS (version 27, IBM Corporation, USA). Demographic and clinical characteristics were compared between groups using independent two-sample *t*-tests for continuous variables, Mann-Whitney U tests for non-normally distributed variables, and chi-square tests for categorical variables.

Tract-based spatial statistics (TBSS) analysis was used to analyze the significant difference clusters of MK values and FA values in the brains of the two groups. Group comparisons of bilateral DTI-ALPS indices, as well as FA and MK values extracted from TBSS-derived regions of interest (ROIs), were conducted using analysis of covariance (ANCOVA) with age and gender as covariates. Effect sizes were reported as partial eta-squared (η^2^). Partial correlation analyses, controlling for age, gender, and education, were performed to examine the associations between: (i) ALPS indices and neuropsychological scores; (ii) ALPS indices and FA/MK values of significant ROIs; and (iii) FA/MK values and neuropsychological scores. To correct for multiple comparisons, the false discovery rate (FDR) was applied, and a corrected P_FDR < 0.05 was considered statistically significant. In this study, the primary analyses focused on the DTI-ALPS index (group comparison and its correlation with cognition and emotion), while secondary analyses explored white matter metrics (FA/MK) using TBSS-derived ROIs.

## 3 Results

### 3.1 Participant characteristics

This study collected data from 56 subjects, including 35 AD patients (19 females, 16 males) and 21 healthy controls (10 females, 11 males). The groups were comparable in terms of age, gender, and education level (Table 2).

**TABLE 2:** Demographic and clinical characteristics of participants.

Characteristic	AD (*n* = 35)	HC (*n* = 21)	*P-*value
Age (years)	66.83 ± 10.29	64.24 ± 12.63	0.406
Gender*			0.629
Male	16 (45.7%)	11 (52.4%)	
Female	19 (54.3%)	10 (47.6%)	
Education (years)	8.57 ± 3.93	8.38 ± 3.28	0.190
MMSE^#^	19 (13–21)	26 (24–27)	<0.001
MoCA	14.57 ± 5.82	24.67 ± 2.90	<0.001
HAMD^#^	5 (4–8)	4 (1–5)	<0.001
HAMA^#^	5 (3–6)	2 (1.5–3.5)	<0.001
ADL^#^	16 (14–22)	65 (57.5–75)	<0.001

Data are presented as mean ± SD for normally distributed variables and as median (IQR) for non- normally distributed variables. Group comparisons:

*Chi- square test; ^#^Mann–Whitney U test; otherwise independent two- sample *t*- test. AD, Alzheimer’s disease; HC, healthy control; MMSE, Mini- Mental State Examination; MoCA, Montreal Cognitive Assessment; HAMD, Hamilton Depression Scale; HAMA, Hamilton Anxiety Scale; ADL, Activities of daily living.

### 3.2 Group differences in bilateral DTI-ALPS indices

**FIGURE 4:**
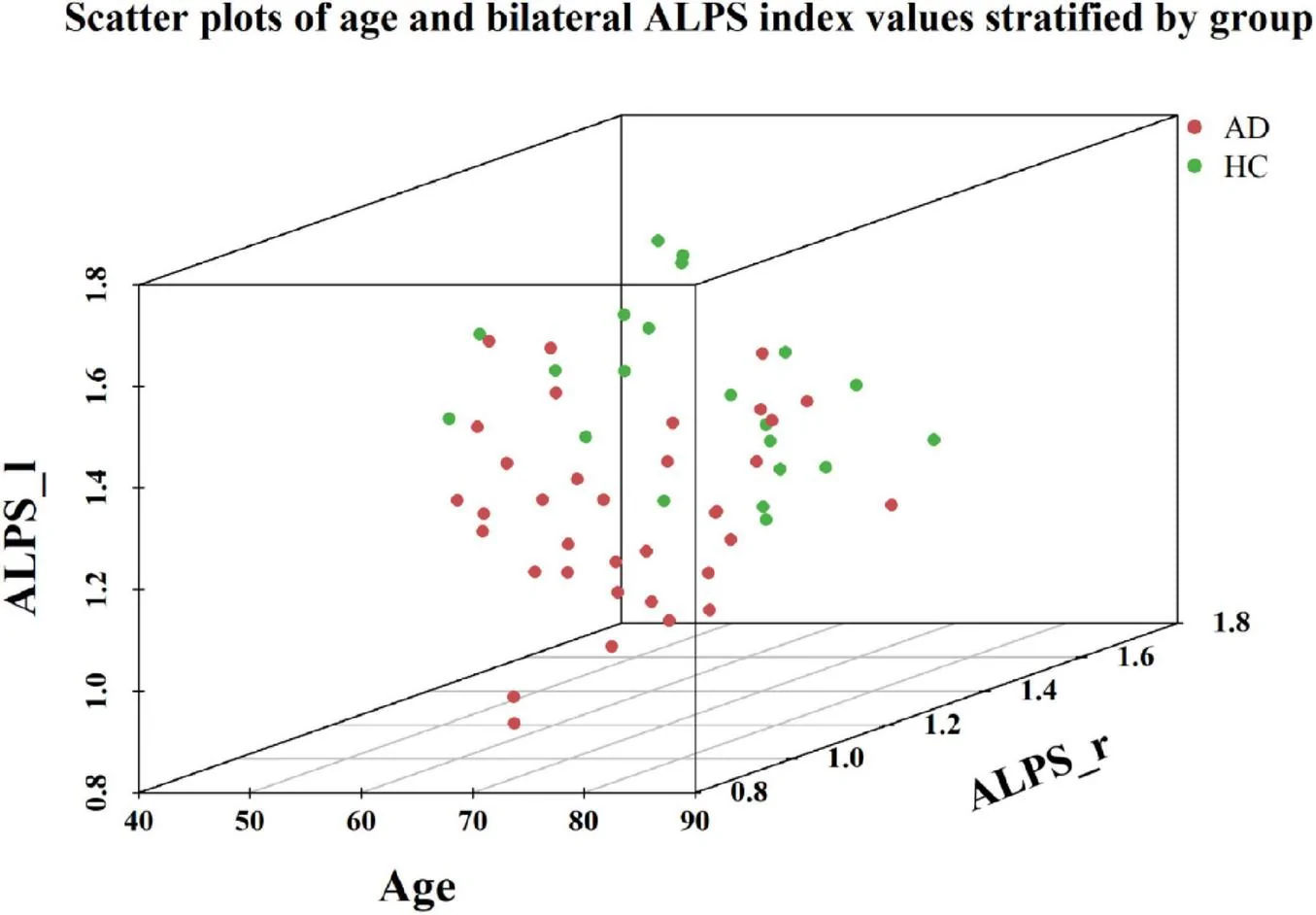
Scatter plots of age and bilateral ALPS index values stratified by group. Three-dimensional scatter plot showing the distribution of individual participants according to age (*x*- axis), right ALPS index (*y*- axis), and left ALPS index (*z*- axis). AD patients are represented by red circles; HC by green circles.

The independent two-sample *t*-test showed that the ALPS index was significantly lower in the AD group than in the HC group for both hemispheres (left: *t* = −4.272, *P* < 0.001; right: *t* = −5.625, *P* < 0.001), indicating a pronounced reduction in glymphatic function in AD patients. Figure 4 summarizes the scatter plot of the bilateral ALPS index values for the two groups of patients. The plot illustrates that AD patients tend to cluster at older ages with lower bilateral ALPS values, whereas controls are generally younger and exhibit higher ALPS indices.

To exclude potential confounding effects of age and gender, we performed ANCOVA on the bilateral ALPS indices with age and gender as covariates. For the ALPS_l index, ANCOVA revealed a significant main effect of group (*F* = 14.404, *P* < 0.001, partial η^2^ = 0.217). The estimated marginal mean (adjusted for age and gender) was 1.236 ± 0.021 for the AD group and 1.369 ± 0.027 for the HC group (Table 3). Age was a significant covariate (*P* < 0.001), while gender was not (*P* = 0.101). For the ALPS_r index, the group difference was also highly significant (*F* = 28.513, *P* < 0.001, partial η2 = 0.354). The adjusted mean was 1.202 ± 0.020, in AD patients and 1.381 ± 0.026, in HC subjects. Both age (*P* < 0.001) and gender (*P* = 0.030) emerged as significant covariates. These results indicate that after controlling for demographic differences, AD patients still exhibit significantly lower bilateral ALPS indices compared to healthy controls, suggesting impaired glymphatic clearance function in AD.

**TABLE 3:** Estimated marginal means of bilateral ALPS indices.

Index	Group	Mean (SE)	95% CI	*F*	*P-*value	Partial η^2^
ALPS_l	AD	1.236 (0.021)	1.194–1.279	14.404	<0.001	0.217
	HC	1.369 (0.027)	1.314–1.425			
ALPS_r	AD	1.202 (0.020)	1.161–1.242	28.513	<0.001	0.354
	HC	1.381 (0.026)	1.328–1.434			

Values are estimated marginal means (adjusted for age and gender) with standard error (SE) and 95% confidence interval. ANCOVA results (F, P, partial η^2^) for the group effect are reported in the last three columns.

### 3.3 Associations between ALPS index and cognitive, functional, and emotional measures

**FIGURE 5:**
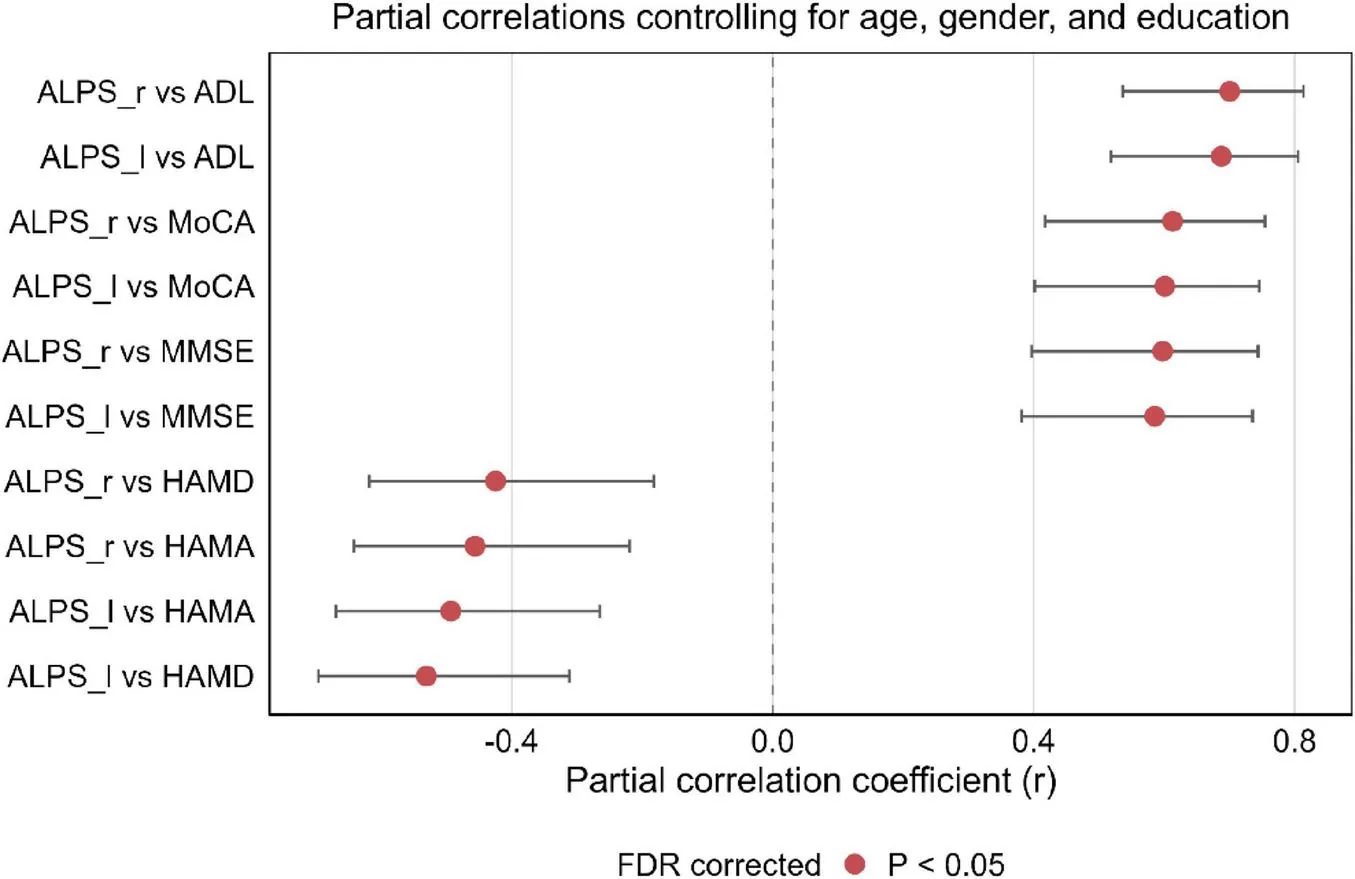
Forest plot of partial correlations between ALPS index and cognitive, functional, and emotional measures. Red points: P_FDR < 0.05; error bars: 95% CI. All associations are significant and directionally consistent.

Partial correlation analyses were performed to examine the associations between bilateral ALPS indices and cognitive, functional, and emotional measures, controlling for age, gender, and education. Multiple comparisons were corrected with the FDR procedure, and P_FDR < 0.05 was considered significant. As shown in Figure 5, both left and right ALPS indices were significantly correlated with cognitive and functional measures, including MMSE, MoCA, and ADL, as well as with emotional measures, including HAMA and HAMD. Specifically, left ALPS showed strong positive correlations with MMSE (*r* = 0.586, P_FDR = 6.8 × 10–6), MoCA (*r* = 0.601, P_FDR = 4.6 × 10–6), and ADL (*r* = 0.688, P_FDR = 6.4 × 10–8), and negative correlations with HAMA (*r* = −0.493, P_FDR = 2.2 × 10–4) and HAMD (*r* = −0.531, P_FDR = 6.2 × 10–5). Right ALPS yielded similar results: positive correlations with MMSE (*r* = 0.598, P_FDR = 4.6 × 10–6), MoCA (*r* = 0.613, P_FDR = 3.5 × 10–6), ADL (*r* = 0.701, P_FDR = 5.2 × 10–8), and negative correlations with HAMA (*r* = −0.456, P_FDR = 6.6 × 10–4) and HAMD (*r* = −0.425, P_FDR = 1.5 × 10–3). All correlations remained significant after FDR correction.

### 3.4 Voxel-wise TBSS analysis of FA and MK

**FIGURE 6:**
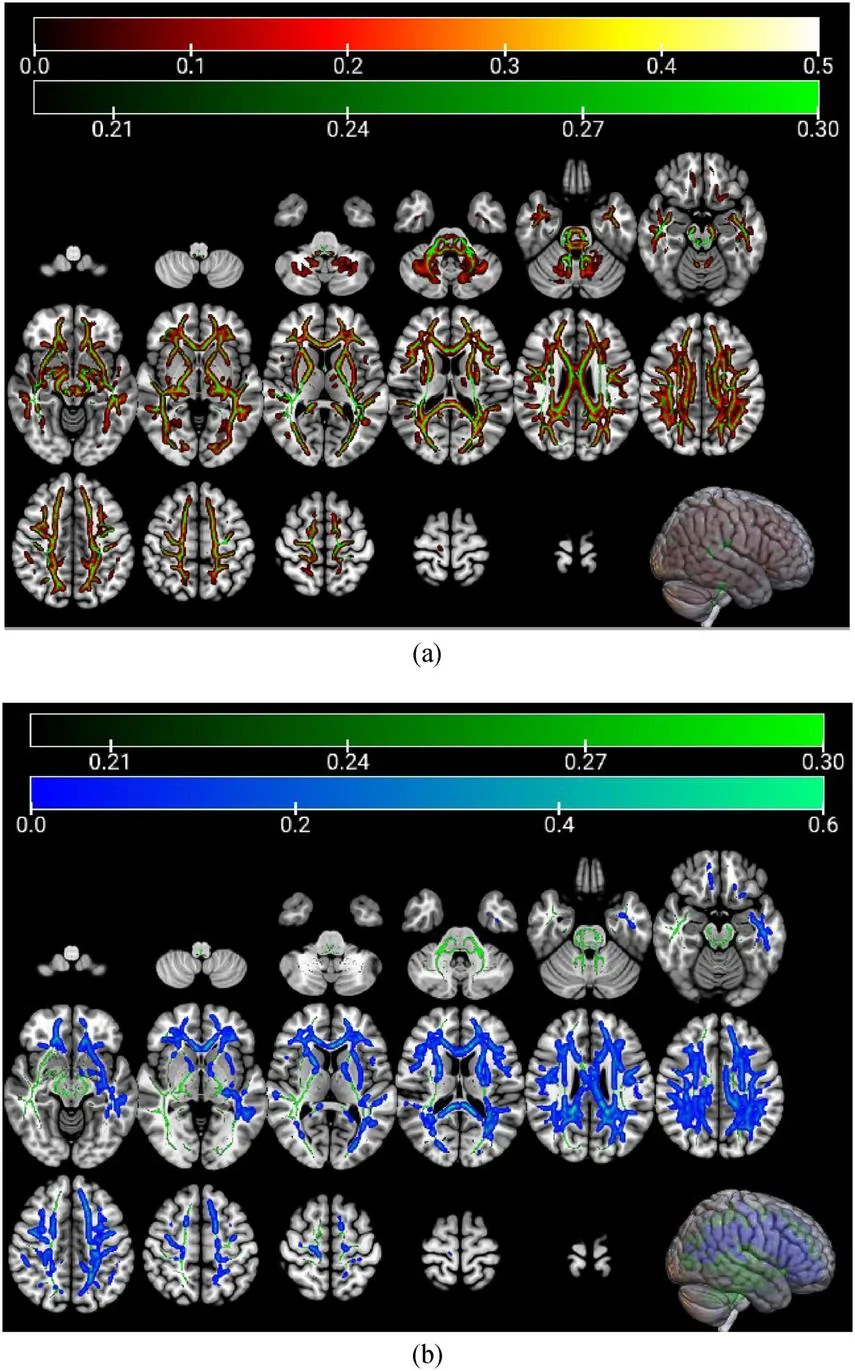
Voxel-wise TBSS results of FA and MK between the AD and HC groups. **(a)** Regions showing significant FA differences between the AD and HC groups. **(b)** Regions showing significant MK differences between the AD and HC groups.

To further explore the structural basis underlying the observed ALPS alterations, we performed voxel-wise comparisons of fractional anisotropy (FA) and mean kurtosis (MK) between the AD and HC groups using TBSS with TFCE correction. Significant differences in white matter microstructure were identified. Specifically, compared with the HC group, the AD group exhibited two clusters with significantly reduced FA and one cluster with significantly reduced MK. The spatial distribution of these significant clusters is shown in Figure 6, and the corresponding peak coordinates and statistical values are summarized in Table 4.

**TABLE 4:** Significant clusters identified by TBSS analysis of FA and MK.

Cluster	Voxels	MNI (X)	MNI (Y)	MNI (Z)	*P*
FA1	74737	82	99	35	0.001
FA2	86	100	83	102	0.048
MK1	37958	114	144	54	0.004

Values represent cluster size (voxels), peak coordinates in MNI space, and corrected *P*-value. One significant MK cluster and two significant FA clusters were identified.

The significant FA cluster was widely distributed across multiple white matter tracts, predominantly involving the forceps minor, superior longitudinal fasciculus, inferior fronto-occipital fasciculus, and anterior thalamic radiation. The MK cluster mainly involved projection and association fibers, including the anterior thalamic radiation and superior longitudinal fasciculus.

To facilitate anatomical interpretation and subsequent analyses, ROI values were extracted from anatomically defined white matter tracts (e.g., hippocampal cingulum and corticospinal tract) based on the significant TBSS clusters.

### 3.5 ROI-based ANCOVA validation of TBSS findings

Consistent with the TBSS findings, ROI-based ANCOVA analyses further supported widespread microstructural alterations in white matter after adjusting for age, gender, and education.

For FA, significant group differences that survived FDR correction were primarily observed in the left hippocampal cingulum and pontine crossing tract, with higher FA values in the HC group compared to the AD group. For MK, a significant difference after FDR correction was identified in the left hippocampal cingulum, while other regions showed similar trends but did not survive multiple comparison correction (Table 5).

**TABLE 5:** ROI-based ANCOVA results for FA and MK values.

Region	FA (*F*)	FA *P*_FDR	FA direction	MK (*F*)	MK *P*_FDR	MK direction
Hippocampal cingulum (L)	11.77	0.0115	HC > AD	13.06	0.017	HC > AD
Pontine crossing tract	11.31	0.0115	HC > AD	\	\	Not significant
Corticospinal tract (L)	2.47	0.1269	HC > AD (trend)	1.93	0.269	HC > AD (trend)
Corticospinal tract (R)	3.16	0.0951	HC > AD (trend)	1.41	0.309	HC > AD (trend)

Values were derived from ANCOVA models adjusted for age, gender, and education. *P-*values were corrected using the FDR method. “Trend” indicates consistent effects that did not survive multiple comparison correction.

### 3.6 Associations between white matter metrics and ALPS index

Based on the TBSS results, ROI values from anatomically defined tracts (e.g., left hippocampal cingulum) were further analyzed. The FA and MK values of fibers within the significant TBSS clusters were extracted for correlation analysis with ALPS index. Partial correlation analyses were performed adjusting for age, gender, and education.

**FIGURE 7:**
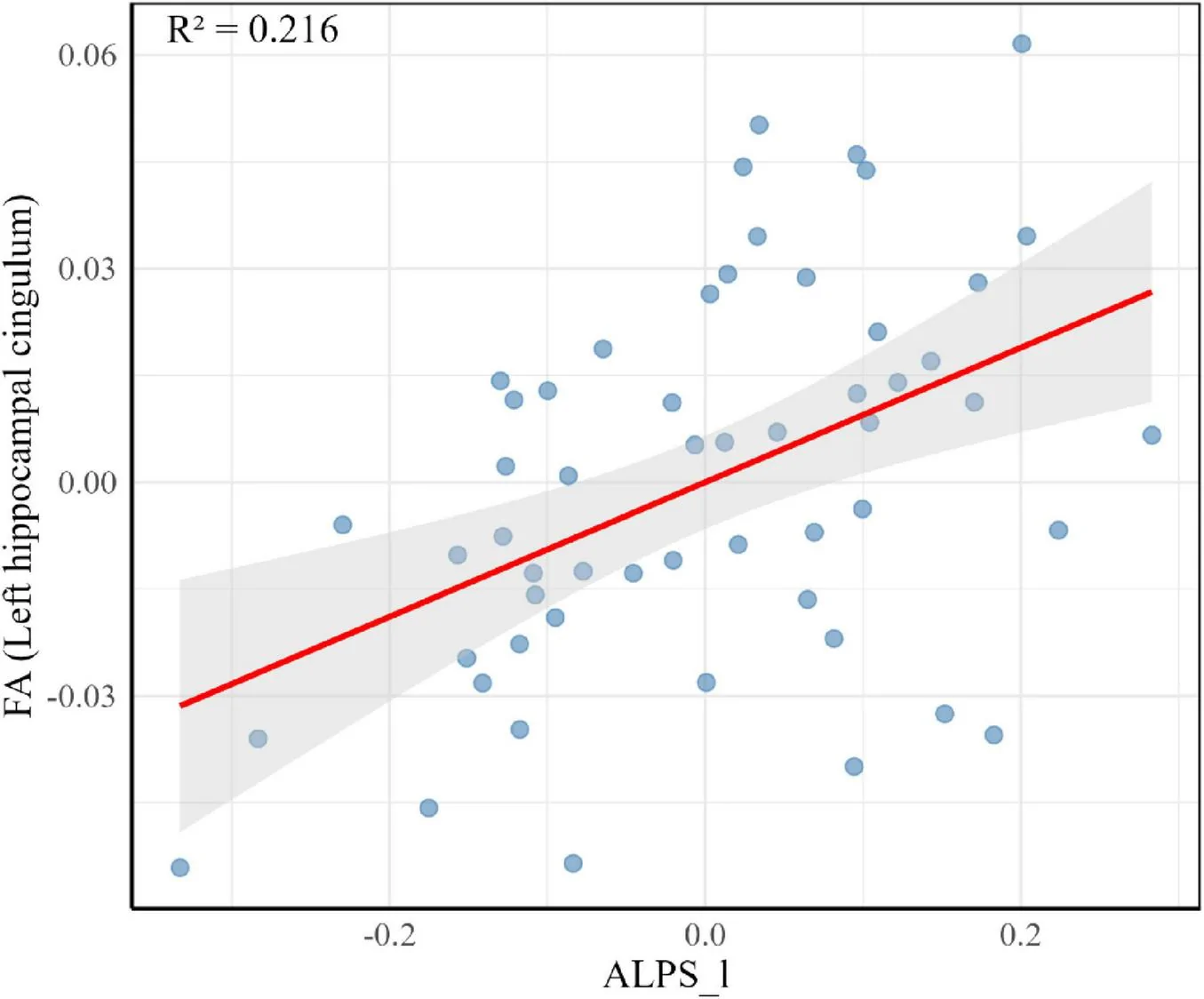
Partial regression plot of the association between left ALPS index and FA of the left hippocampal cingulum. Partial regression plot adjusted for age, gender, and education. The red line represents the linear regression fit (R^2^ = 0.216), with the gray band indicating the 95% confidence interval.

As shown in Table 6, the left ALPS index was significantly positively correlated with FA of the left hippocampal cingulum (*r* = 0.465, P_FDR = 0.0020) and with MK of the left hippocampal cingulum (*r* = 0.441, P_FDR = 0.0033). Similarly, significant positive correlations were observed between left ALPS and MK of the left (*r* = 0.516, P_FDR = 0.0005) and right (*r* = 0.491, P_FDR = 0.0010) corticospinal tracts. Notably, no negative correlations survived FDR correction. Figure 7 illustrates the partial regression plot for the most robust association, i.e., between left ALPS and FA of the left hippocampal cingulum, after removing the effects of age, gender, and education. The scatter plot of residuals confirms a clear positive linear relationship, with the fitted line explaining 21.6% of the variance (R^2^ = 0.216). This indicates that better glymphatic function (higher ALPS) is associated with greater white matter integrity (higher FA and MK) in these regions. These findings indicate that higher ALPS (better glymphatic function) is associated with higher FA and MK in several key white matter regions, suggesting that preserved white matter microstructural integrity may accompany more efficient glymphatic clearance in AD patients.

**TABLE 6:** Partial correlations of white matter metrics with left ALPS.

White matter region	Metric	Partial r	*P*	*P_*FDR
Hippocampal cingulum (L)	FA	0.465	5.1 × 10^–4^	2.0 × 10^–3^
Hippocampal cingulum (L)	MK	0.441	0.001	3.3 × 10^–3^
Corticospinal tract (L)	MK	0.516	9.0 × 10^–5^	5.0 × 10^–4^
Corticospinal tract (R)	MK	0.491	2.2 × 10^–4^	1.0 × 10^–3^

All partial correlations controlled for age, gender, and education. Only part of the FDR corrected significant results are displayed.

### 3.7 Associations between white matter metrics and cognitive and functional measures

**FIGURE 8:**
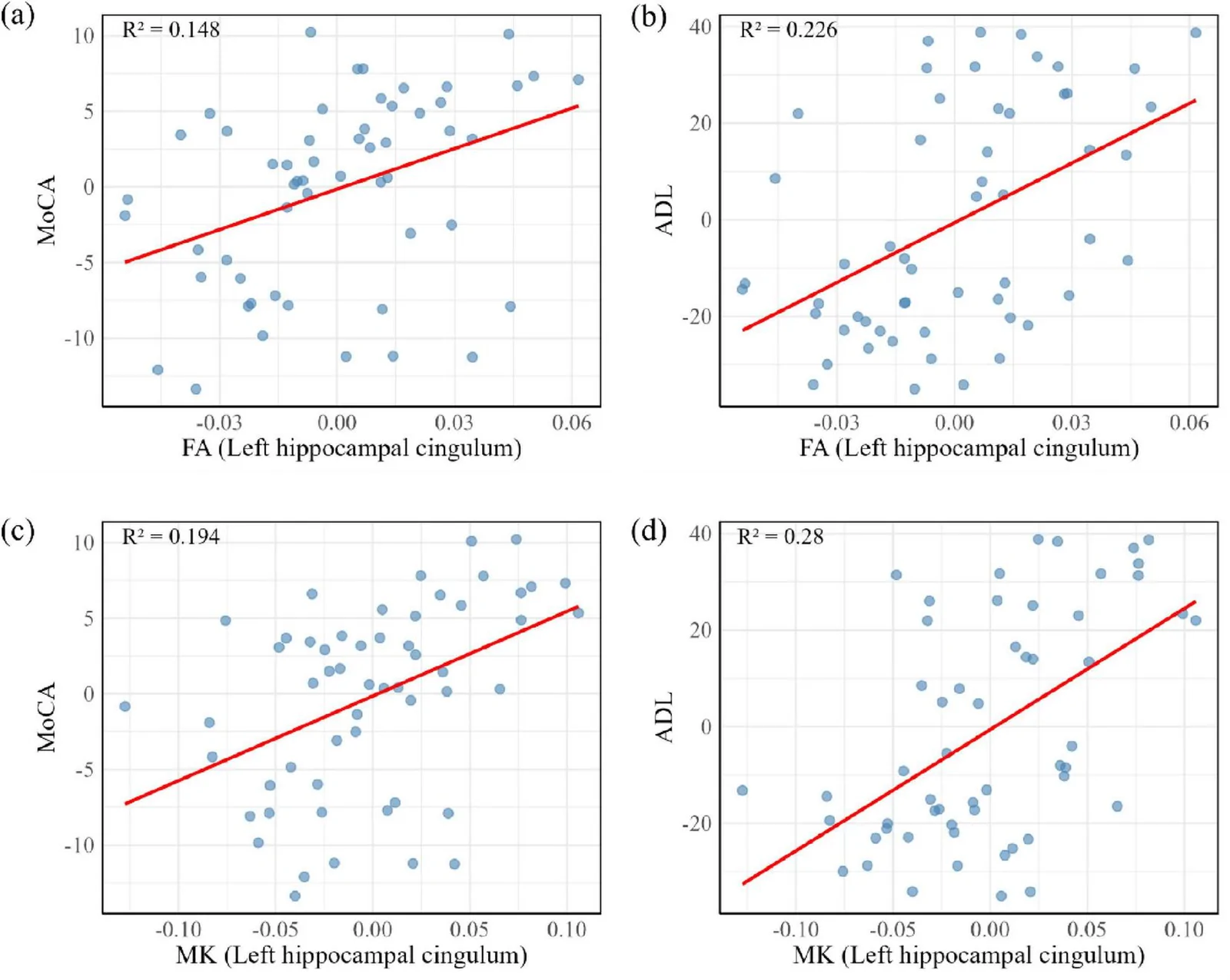
Partial regression plots of white matter metrics of the left hippocampal cingulum against cognitive and functional measures. **(a)** FA vs. MoCA; **(b)** FA vs. ADL; **(c)** MK vs. MoCA; **(d)** MK vs. ADL. Each dot represents one participant. Red lines indicate linear regression fits. *R*^2^ values are displayed in each panel.

Partial correlation analysis, after controlling for age, gender, and education, showed that the FA value of the left hippocampal cingulum was significantly positively correlated with MoCA (*r* = 0.385, *P* = 0.005) and ADL (*r* = 0.476, *P* < 0.001). Similarly, the MK value of the left hippocampal cingulum was significantly positively correlated with MoCA (*r* = 0.441, *P* = 0.001) and ADL (*r* = 0.529, *P* < 0.001). As shown in Figure 8, the scatter plots illustrate these positive relationships after adjusting for covariates.

## 4 Discussion

In this study, we primarily demonstrated that the bilateral ALPS indices were significantly reduced in patients with AD compared with healthy controls, and these differences remained significant after adjustment for age and gender. We further showed that lower ALPS indices were significantly associated with worse cognitive performance, poorer functional status, and more severe emotional symptoms after controlling for age, gender, and education, with all associations remaining significant after FDR correction. As secondary analyses, we performed TBSS to identify white matter damage regions in AD patients and examined their associations with cognition and the ALPS index. We identified widespread white matter microstructural alterations using TBSS and ROI-based analyses, suggesting that white matter abnormalities may provide a structural substrate underlying glymphatic dysfunction in AD. These findings support the view that impaired glymphatic clearance is closely linked to the clinical manifestations of AD.

Previous studies have analyzed various neurodegenerative diseases such as AD, Parkinson’s disease, frontotemporal dementia, diabetic encephalopathy, delirium, and narcolepsy using the ALPS index in relation to chronic brain injury (Gumeler et al., 2023; Jiang et al., 2023; Shen et al., 2022; Taoka et al., 2017; Tu et al., 2024). Other articles have suggested that a decrease in the ALPS index is associated with clinical motor abilities and cognitive scores in Parkinson’s disease patients, and there are also studies indicating that cognitive decline in diabetic encephalopathy patients is related to the ALPS index (Costa et al., 2024; Yu et al., 2024). Similar to frontotemporal dementia and diabetic encephalopathy, AD shares similar pathological mechanisms, including neuronal degeneration, neuroinflammation, and chronic oxidation. In a study conducted last year based on the UKB and ADNI databases, results showed that the ALPS index in AD patients decreased, which is associated with the progression from mild cognitive impairment to AD and exhibited a correlation with amyloid deposition in PET scans. The ALPS index declined as early as the preclinical and prodromal stages of AD, indicating that glymphatic failure or damage may occur in the early stages of AD. Furthermore, glymphatic dysfunction may even occur prior to amyloid pathology. Changes in glymphatic activity occurred even before the increase in Aβ PET burden and before CSF Aβ42 reached the positive threshold (Huang et al., 2024). This also effectively corroborates the results of our current experiment.

As early as 2017, scholars proposed the concept of ALPS and used this method to evaluate AD patients (Taoka et al., 2017). In the subsequent years up to the present, multiple scholars have utilized the ALPS index to analyze various neurodegenerative diseases and obtained positive results, demonstrating that the ALPS index can reflect the clearance function of the brain’s glymphatic system (Bae et al., 2023; Hong et al., 2024; Yin et al., 2024; Zhang et al., 2022, 2023, 2024). However, some scholars have raised questions about the extent to which the ALPS index serves as a marker of glymphatic function, which remains a topic of active debate. The main criticisms of this technology include that the signal is not particularly sensitive to cerebrospinal fluid, there is insufficient evidence to prove that the measured signal is limited to PVS, and that the visible PVS adjacent to the external ventricles is not perivenous (Agarwal et al., 2024). Preclinical studies have found specific regional differences in clearance rates of glymphatic pathways, with some studies anatomically dividing the ALPS index into multiple regions (Ran et al., 2025). Existing studies have demonstrated that the glymphatic system aids in the clearance of amyloid proteins in the brains of mice (Iliff et al., 2012). Therefore, considering the existence of this situation, this study did not take the ALPS index of the whole brain but rather the separate ALPS indices of the left and right hemispheres, as the overall brain composite index may not reflect the different conditions of the left and right hemispheres. In this study, it was also confirmed that the changes in the ALPS indices of the left and right hemispheres are associated with different manifestations of cognition and white matter injury. The relationship between the ALPS index and the function of the human glymphatic system has not been conclusively or rigorously verified through pathophysiological research; therefore, the ALPS index should be regarded as an indirect imaging proxy of glymphatic function rather than a direct measurement. This index requires further validation of its authenticity and validity through pathophysiological experiments. For AD patients, cognitive changes are a core symptom. The decline of the ALPS index is not a feature unique to dementia patients and can be found in various other neurodegenerative diseases. Although the ALPS index is sensitive in detecting cognitive impairment, it lacks the specificity to differentiate these conditions, which is another limitation of the index that still requires further research.

After controlling for age, gender, and education, we found that both left and right ALPS indices were significantly correlated with all cognitive (MMSE, MoCA), functional (ADL), and emotional (HAMA, HAMD) measures. These findings indicate that glymphatic dysfunction in AD is not only linked to cognitive decline, but is also related to functional impairment and emotional burden. Although both hemispheres showed significant associations, the right ALPS index showed slightly stronger correlations with ADL and MoCA, suggesting possible hemispheric differences that merit further investigation. All correlation analyses were corrected for multiple comparisons using the FDR method. The fact that nearly all associations remained significant at *P* < 0.005 indicates that the observed relationships are highly robust and unlikely to be false positives. The inclusion of gender as an additional covariate and the use of FDR correction further strengthened the observed correlations, underscoring the importance of rigorous statistical adjustment.

In previous studies, there have also been analyses of the correlation between the ALPS index and the FA and MD values obtained from whole-brain DTI (Lee et al., 2022). Unlike other studies, this research also included TBSS analysis and explored the correlation of its results with the ALPS index. Due to the relative complexity of human brain cells and the interference of organelles, the diffusion of water molecules is strictly speaking non-Gaussian, and DTI has disadvantages compared to DKI; the tightly interwoven fiber bundles in the brain mean that DKI parameters can better reflect the directionality at their intersection points, thus making the results relatively more accurate. Our secondary analyses provided structural support for the observed ALPS alterations. TBSS revealed widespread white matter microstructural abnormalities in AD, and ROI-based analyses showed that the left hippocampal cingulum exhibited significant group differences in both FA and MK after FDR correction. Moreover, left ALPS was positively correlated with both FA and MK in the left hippocampal cingulum, and with MK in the bilateral corticospinal tracts. The left hippocampal cingulum was also positively associated with MoCA and ADL. Taken together, these results suggest that preserved white matter microstructural integrity, particularly in the left hippocampal cingulum, may accompany more efficient glymphatic clearance and better cognitive and functional performance in AD. Although the AD group showed a higher mean age, this difference was partly affected by extreme values, whereas the median age difference was less marked. In addition, age was further adjusted for in the subsequent analyses, supporting the robustness of the main findings.

Certainly, this study also has its limitations. Firstly, due to the strict inclusion criteria, the sample size of this study is relatively small, and the patient group consists entirely of East Asian individuals, which may not reflect the performance of the entire human population. Furthermore, we did not group patients based on cognitive scores, thus the performance of the ALPS index among AD patients with different cognitive levels remains unexplored. In addition, the ROI-based tract analyses were derived from TBSS-identified regions and should therefore be interpreted as secondary validation analyses rather than independent whole-brain findings. Although multiple comparisons were controlled using the FDR method, the modest sample size may still limit the stability of tract-level estimates. Again, we generated DTI images by splitting DKI with *b* = 1000 s/mm^2^, without exploring other different *b*-values to calculate the ALPS index. A study has indicated that different scanning parameters can significantly impact the results (Taoka and Naganawa, 2021). Finally, since this study is cross-sectional, there is no longitudinal research on the prognosis of patients and the changes in their ALPS indices, which warrants further investigation.

## 5 Conclusion

Patients with AD showed reduced bilateral DTI-ALPS indices, suggesting impaired glymphatic clearance function. Lower ALPS indices were associated with worse cognitive, functional, and emotional measures. Secondary analyses further suggested that white matter abnormalities, especially in the left hippocampal cingulum, may be linked to glymphatic dysfunction in AD.

## Data Availability

The original contributions presented in this study are included in this article/supplementary material, further inquiries can be directed to the corresponding authors.
